# Supplementary material for: Nonlocality, integrability and quantum chaos in the spectrum of Bell operators
Source: npj Quantum Inf. 2026 Apr 6;12(1):120. doi: 10.1038/s41534-026-01232-z (PMC13421339; doi:10.1038/s41534-026-01232-z)
Supplement: Supplementary file 1 — Supplementary information [file 41534_2026_1232_MOESM1_ESM.pdf]

Supplementary Material for "Nonlocality, Integrability and Quantum Chaos in the Spectrum of Bell Operators"

Albert Aloy (corresponding author),[1,2,*] Guillem Müller-Rigat,[3] Maciej Lewenstein,[3,4] Jordi Tura,[5,6] and Matteo Fadel (corresponding author)[7,†]

[1] *Institute for Quantum Optics and Quantum Information, Austrian Academy of Sciences, Boltzmanngasse 3, A-1090 Vienna, Austria*
[2] *Vienna Center for Quantum Science and Technology (VCQ), Faculty of Physics, University of Vienna, Vienna, Austria*
[3] *ICFO-Institut de Ciencies Fotoniques, The Barcelona Institute of Science and Technology, Castelldefels (Barcelona) 08860, Spain.*
[4] *ICREA, Pg. Lluís Companys 23, 08010 Barcelona, Spain.*
[5] *$\langle aQa^L\rangle$ Applied Quantum Algorithms, Universiteit Leiden*
[6] *Instituut-Lorentz, Universiteit Leiden, P.O. Box 9506, 2300 RA Leiden, The Netherlands*
[7] *Department of Physics, ETH Zürich, 8093 Zürich, Switzerland*

SUPPLEMENTARY NOTE 1: CLASSICAL BOUND FOR THE BELL INEQUALITY (1) IN THE MAIN TEXT

Here we provide a proof showing that the Bell inequality B introduced in the main text has classical bound $\beta_c = 0$ for any number of parties n. That is, we want to show that

$$B = (\mathcal{P}_{0|0} + \mathcal{P}_{0|1} + \mathcal{P}_{1|0} + \mathcal{P}_{1|1}) + (\mathcal{P}_{00|00} + \mathcal{P}_{00|11} + \mathcal{P}_{11|00} + \mathcal{P}_{11|11}) - 2(\mathcal{P}_{01|01} + \mathcal{P}_{01|10}) \geq 0, \tag{1}$$

where recall that $\mathcal{P}_{a|x} = \sum_{i\in[n]} p_i(a|x)$ is the collective one-body conditional probability, with $p_i(a|x)$ denoting the probability that subsystem i yields outcome a given measurement setting x. Similarly, $\mathcal{P}_{ab|xy} = \sum_{i\neq j\in[n]} p_{ij}(ab|xy)$ represents the collective two-body conditional probability summing over all possible pairs $i \neq j \in [n]$.

First, in order to account for all local hidden variables, we want to come up with a parametrization to describe the conditional probabilities in terms of Local Deterministic Strategies (LDS). Because our Bell inequality is permutationally invariant, many Bell inequality coefficients take the same values at many LDSs, which leads to redundancies. Hence, instead of considering all the d^{mn} possibilities of the general case (3^{2n} for our case with 3 outcomes an 2 measurements), we propose the following (much more efficient) parametrization: Suppose that at each run all the outcomes for each possible measurement and party are predetermined. Then, let $c_{a,a'}$ be the total number of parties that have predetermined the pair of outcomes $a, a' \in \{0, 1, 2\}$ for the two possible measurement settings $x \in \{0, 1\}$ respectively. It follows by definition that $c_{a,a'} \geq 0$ and $\sum_{a,a'} c_{a,a'} = n$. Therefore, following this parametrization, the symmetrized one-body conditional probabilities $\mathcal{P}_{a|x}$ under a given LDS can be expressed as:

$$\mathcal{P}_{a|x} := \sum_{i=1}^{n} p_i(a|x) \overset{\text{LDS}}{=} \begin{cases} c_{a,0} + c_{a,1} + c_{a,2} & \text{for } x = 0 \\ c_{0,a} + c_{1,a} + c_{2,a} & \text{for } x = 1 \end{cases} . \tag{2}$$

On the other hand, the symmetric two-body conditional probabilities $\mathcal{P}_{ab|xy}$ factorize under a given LDS as:

$$\begin{aligned} \mathcal{P}_{ab|xy} &:= \sum_{i\neq j} p_{ij}(ab|xy) \overset{\text{LDS}}{=} \sum_{i\neq j} p_i(a|x) p_j(b|y) \\ &= \underbrace{\sum_{i\neq j} p_i(a|x) p_j(b|y) + \sum_{i} p_i(a|x) p_i(b|y)}_{\mathcal{P}_{a|x}\cdot\mathcal{P}_{b|y}} - \underbrace{\sum_{i} p_i(a|x) p_i(b|y)}_{:=Q_{ab|xy}} , \end{aligned} \tag{3}$$

where we have defined

$$Q_{ab,xy} := \begin{cases} \mathcal{P}_{a|x} & \text{if } a = b, x = y \\ 0 & \text{if } a \neq b, x = y \\ c_{a,b} & \text{if } x = 0, y = 1 \\ c_{b,a} & \text{if } x = 1, y = 0 \end{cases} . \tag{4}$$

* albert.aloy@oeaw.ac.at
† fadelm@phys.ethz.ch

Note that one can neglect one of the outcomes without loss of generality by means of the NS principle,

$$P(a_1,\dots,\hat{a}_i,\dots,a_n|x_1,\dots,\hat{x}_i,\dots,x_n) \equiv \sum_{a_i\in\{0,1,2\}} P(a_1,\dots,a_n|x_1,\dots,x_n)\ ,$$

where $\hat{\cdot}$ denotes the absence of that coordinate and the $\equiv$ symbol means that the LHS of Eq. (5) is well-defined; *i.e.*, it does not depend on the value of x_i. Hence, for instance we can take Eqs. (2) to (4) with $a, b \in \{0,1\}$. Notice also that it is straightforward to generalize our parametrization to any number of outcomes d.

Finally, in Tab. I we express the one-body terms and the factorized two-body terms as a function of the quantities $c_{a,a'}$. Therefore, all the possible local-realist correlations in a $(n,2,3)$ Bell-type experiments can be described in terms of the relations in Supplementary Table I and shared randomness. Moreover, the local polytope for an $(n,2,3)$ permutationally invariant Bell scenario characterized by one- and two-body correlators is formed by the convex hull of all the configurations satisfying the relations in Supplementary Table I.

$\mathcal{P}_{0\|0} \overset{\text{LDS}}{=} c_{0,0}+c_{0,1}+c_{0,2}$	$\mathcal{P}_{00\|00} \overset{\text{LDS}}{=} \mathcal{P}_{0\|0}^2 - \mathcal{P}_{0\|0}$	$\mathcal{P}_{00\|01} \overset{\text{LDS}}{=} \mathcal{P}_{0\|0}\mathcal{P}_{0\|1} - c_{0,0}$	$\mathcal{P}_{00\|10} \overset{\text{LDS}}{=} \mathcal{P}_{00\|01}$	$\mathcal{P}_{00\|11} \overset{\text{LDS}}{=} \mathcal{P}_{0\|1}^2 - \mathcal{P}_{0\|1}$
$\mathcal{P}_{1\|0} \overset{\text{LDS}}{=} c_{1,0}+c_{1,1}+c_{1,2}$	$\mathcal{P}_{01\|00} \overset{\text{LDS}}{=} \mathcal{P}_{0\|0}\mathcal{P}_{1\|0}$	$\mathcal{P}_{01\|01} \overset{\text{LDS}}{=} \mathcal{P}_{0\|0}\mathcal{P}_{1\|1} - c_{0,1}$	$\mathcal{P}_{01\|10} \overset{\text{LDS}}{=} \mathcal{P}_{10\|01}$	$\mathcal{P}_{01\|11} \overset{\text{LDS}}{=} \mathcal{P}_{0\|1}\mathcal{P}_{1\|1}$
$\mathcal{P}_{0\|1} \overset{\text{LDS}}{=} c_{0,0}+c_{1,0}+c_{2,0}$	$\mathcal{P}_{10\|00} \overset{\text{LDS}}{=} \mathcal{P}_{01\|00}$	$\mathcal{P}_{10\|01} \overset{\text{LDS}}{=} \mathcal{P}_{1\|0}\mathcal{P}_{0\|1} - c_{1,0}$	$\mathcal{P}_{10\|10} \overset{\text{LDS}}{=} \mathcal{P}_{01\|01}$	$\mathcal{P}_{10\|11} \overset{\text{LDS}}{=} \mathcal{P}_{01\|11}$
$\mathcal{P}_{1\|1} \overset{\text{LDS}}{=} c_{0,1}+c_{1,1}+c_{2,1}$	$\mathcal{P}_{11\|00} \overset{\text{LDS}}{=} \mathcal{P}_{1\|0}^2 - \mathcal{P}_{1\|0}$	$\mathcal{P}_{11\|01} \overset{\text{LDS}}{=} \mathcal{P}_{1\|0}\mathcal{P}_{1\|1} - c_{1,1}$	$\mathcal{P}_{11\|10} \overset{\text{LDS}}{=} \mathcal{P}_{11\|01}$	$\mathcal{P}_{11\|11} \overset{\text{LDS}}{=} \mathcal{P}_{1\|1}^2 - \mathcal{P}_{1\|1}$

Supplementary Table I. Resulting one- and two-body conditional probabilities under an LDS.

Now that we have a parametrization to incorporate the LDSs into the conditional probabilities, we are ready to substitute the corresponding conditional probabilities in the Bell inequality. After rearranging the terms, one ends up with the following polynomial:

$$\begin{aligned} B = {} & (c_{00}+c_{02})^2 + (c_{00}+c_{20})^2 + (c_{11}+c_{12})^2 + (c_{11}+c_{21})^2 \\ & + (c_{00}-c_{12})^2 + (c_{00}-c_{21})^2 + (c_{11}-c_{02})^2 + (c_{11}-c_{20})^2 \\ & + 2(c_{10}+c_{01}) - 2(c_{00}+c_{11})^2 - (c_{12}+c_{20})^2 - (c_{02}+c_{21})^2, \end{aligned} \tag{5}$$

where $c_{i,j} \geq 0$ for all $i, j \in \{0,1,2\}$ and they fulfill the constraint $\sum_{0\leq i,j<3} c_{ij} = n$ with n the total number of parties. Notice that the term c_{22} does not appear in the expression, thus we can set any $0 \leq c_{22} \leq n$ without contributing in the classical bound. Thus it is trivial to see that there exists at least one strategy leading to $B = 0$, *i.e.* setting $c_{22} = n$. Consequently, we just have to prove that B cannot take negative values.

Proof that $B \geq 0$: We are interested in the minimal value that (5) can achieve. Since the terms $2(c_{10}+c_{01})$ will always add a positive or zero contribution, we can set them to $c_{10} = c_{01} = 0$ without loss of generality to find the minimal value of B. Therefore we simplify the problem to look at the minimal value of:

$$\begin{aligned} \tilde{B} = {} & (c_{00}+c_{02})^2 + (c_{00}+c_{20})^2 + (c_{11}+c_{12})^2 + (c_{11}+c_{21})^2 \\ & + (c_{00}-c_{12})^2 + (c_{00}-c_{21})^2 + (c_{11}-c_{02})^2 + (c_{11}-c_{20})^2 \\ & - 2(c_{00}+c_{11})^2 - (c_{12}+c_{20})^2 - (c_{02}+c_{21})^2. \end{aligned} \tag{6}$$

After expanding and rearranging the terms we reach the following equivalent polynomial:

$$\begin{aligned} \tilde{B} = 2\Big[& c_{00}^2 + c_{11}^2 + \frac{c_{02}^2+c_{20}^2}{2} + \frac{c_{12}^2+c_{21}^2}{2} \\ & + c_{00}(c_{02}+c_{20}) - c_{11}(c_{02}+c_{20}) + c_{11}(c_{12}+c_{21}) - c_{00}(c_{12}+c_{21}) \\ & - c_{02}c_{21} - c_{12}c_{20} - 2c_{00}c_{11}\Big]. \end{aligned} \tag{7}$$

Then, the condition for (7) to take negative values corresponds to the following inequality

$$c_{11}(c_{02}+c_{20}) + c_{00}(c_{12}+c_{21}) + c_{02}c_{21} + c_{12}c_{20} + 2c_{00}c_{11} > c_{00}^2 + c_{11}^2 + \frac{c_{02}^2+c_{20}^2}{2} + \frac{c_{12}^2+c_{21}^2}{2} + c_{00}(c_{02}+c_{20}) + c_{11}(c_{12}+c_{21})\ , \tag{8}$$

which can be rearranged as:

$$(c_{00}-c_{11})(c_{12}+c_{21}-c_{02}-c_{20}) > (c_{00}-c_{11})^2 + \frac{(c_{02}-c_{21})^2}{2} + \frac{(c_{12}-c_{20})^2}{2}\ . \tag{9}$$

Our goal is to find that such condition leads to a contradiction for all cases in order to show that I cannot take a negative value. First it is convenient to define de variables $x := c_{00}-c_{11}, y := c_{12}-c_{20}, z := c_{02}-c_{21}$, so that the condition gets expressed as:

$$x(y-z) - \left(x^2 + \frac{y^2}{2} + \frac{z^2}{2}\right) > 0. \tag{10}$$

Take now $f(x,y,z) = x(y-z) - (x^2 + \frac{y^2}{2} + \frac{z^2}{2})$ in order to find its critical points $\nabla f(x,y,z) = (-2x+y-z, x-y, -z-x)$, $\nabla f(x^*,y^*,z^*) = 0 \Rightarrow x^* = y^*, z^* = -x^*$, where $f(x^*,y^*,z^*) = 0$. Next, by looking at its Hessian matrix $\boldsymbol{H}(f(x,y,z))$, where $(\boldsymbol{H}(f(x,y,z)))_{ij} = \frac{\partial^2 f}{\partial x_i \partial x_j}$, one sees that the resulting Hessian matrix has eigenvalues $\{-3,-1,0\}$ and therefore it is negative semidefinite. Thus, the critical point corresponds to the maximum.

We conclude that (10) leads to a contradiction for all values of c_{ij} and, consequently, I cannot take negative values. Finally, since the argument is independent of n and we have seen that $I = 0$ is a valid local deterministic strategy, it follows that the classical bound is $\beta_c = 0$ for all n.

□

SUPPLEMENTARY NOTE 2: USING THE NEAREST-NEIGHBOR SPACING DISTRIBUTION (NNSD)

Before adopting the ratio of consecutive level spacings (RCS) as our main chaos indicator, we initially performed our analysis using the traditional nearest-neighbor spacing distribution (NNSD). While NNSD has been extensively used in the literature to probe quantum chaos [1], it requires spectrum unfolding (see below) and is sensitive to binning choices and other parameters, which can introduce ambiguities. Despite these drawbacks, our findings using NNSD and the Brody distribution interpolation are consistent with the conclusions based on RCS presented in the main text.

In this Supplementary Note, for completion and comparison, we provide said NNSD results. Rather than repeating the full analysis, we summarize the key steps and highlight where the NNSD confirms the insights obtained through the RCS method. The methodology is the same as the one presented in the main text, except that in the last step we compute the NNSD of the resulting Bell operator (instead of the RCS) and fit it to the Brody distribution Eq. (11) to extract the parameter ω as we explain in what proceeds.

Fitting to the Brody Distribution.— If the NNSD follows a Poisson distribution, then the NNSD typically indicates "level attraction" in the spectrum, signalling an integrable system in the classical limit. If, on the contrary, the NNSD follows a Wigner-Dyson distribution, then the NNSD typically illustrates "level repulsion", which signals a chaotic system in the classical limit (equivalently described by random matrix theory). For this reason, it is convenient to fit the NNSD computed numerically with the so-called Brody distribution [2, 3], which gives a function interpolating between these two limit cases. In particular, for random matrices sampled from the Gaussian orthogonal ensemble, after unfolding the spectrum within each irreducible representation (irrep) of SU(3), the Brody distribution fit of a level spacing distribution $P(s)$ is [2, 3],

$$P(s,\omega) = A(\omega+1)s^{\omega}\exp\left(-As^{\omega+1}\right), \tag{11}$$

where $A = \left(\Gamma\left(\frac{\omega+2}{\omega+1}\right)\right)^{\omega+1}$ with Γ denoting the gamma function, and $\omega \in [0,1]$ is the Brody parameter interpolating between the Poisson ($\omega = 0$) and Wigner-Dyson ($\omega = 1$) statistics.

PIBI irreducible representations and NNSD.– In Supplementary Figure 1 we show the RCS and NNSD results for $n = 25$, along with the corresponding interpolating parameters λ and ω displayed under each irrep for comparison. For better readability, these values are also listed in Supplementary Table II. As discussed in the main text, Supplementary Figure 1 indicates whether the RCS and NNSD of a given irrep are characteristic of a Poissonian distribution (blue, $\lambda = 0$ & $\omega = 0$, signalling integrability) or a Wigner-Dyson distribution (orange, $\lambda > 0$ & $\omega > 0$, signalling chaos).

We exclude irreps that do not exhibit nonlocality detection (*i.e.,* those for which $\langle\mathcal{B}\rangle \geq 0$ for all measurement choices) from our analysis, since such cases allow for trivial strategies saturating the classical bound $\langle\mathcal{B}\rangle = 0$ independently of the irrep and do not provide a meaningful setting to probe the relation between nonlocality and spectral statistics. In particular, one such strategy consists in having the same measurement settings in both input possibilities (*i.e.,* $\boldsymbol{\theta}_0 = \boldsymbol{\theta}_1$).

One observes that, even though the NNSD results are in principle less robust and dependent on the unfolding procedure, the same behavior is observed across both analysis. That is, when restricting to the optimal measurements to obtain the lowest

Irrep	RCS λ	NNSD (Brody) ω	$\langle\mathcal{B}\rangle$
(9,8)	4.559×10^{-1}	3.217×10^{-1}	−0.393
(14,1)	2.397×10^{-8}	3.754×10^{-12}	6.939×10^{-10}
(13,3)	3.092×10^{-7}	3.229×10^{-11}	−0.231
(12,5)	2.941×10^{-7}	3.347×10^{-11}	−0.386
(11,7)	2.692×10^{-7}	4.241×10^{-11}	−0.736
(16,0)	7.240×10^{-8}	4.845×10^{-11}	−0.366
(15,2)	1.098×10^{-1}	1.723×10^{-1}	−0.468
(14,4)	3.208×10^{-7}	3.076×10^{-11}	−0.809
(13,6)	7.680×10^{-10}	6.352×10^{-11}	−1.065
(17,1)	9.281×10^{-8}	4.574×10^{-11}	−0.914
(16,3)	6.129×10^{-10}	3.922×10^{-8}	−1.152
(15,5)	2.936×10^{-7}	2.977×10^{-9}	−1.522
(19,0)	5.987×10^{-8}	4.606×10^{-8}	−1.334
(18,2)	1.518×10^{-7}	7.226×10^{-9}	−1.668
(17,4)	1.984×10^{-7}	3.889×10^{-11}	−1.991
(20,1)	1.575×10^{-7}	2.985×10^{-7}	−2.180
(19,3)	7.193×10^{-9}	4.232×10^{-9}	−2.543
(22,0)	8.086×10^{-8}	2.691×10^{-11}	−2.786
(21,2)	1.044×10^{-8}	1.153×10^{-9}	−3.137
(23,1)	1.144×10^{-10}	1.105×10^{-9}	−3.800
(25,0)	6.126×10^{-8}	2.903×10^{-11}	−4.522

Supplementary Table II. The interpolating numbers obtained for the RCS and NNSD, together with the corresponding nonlocality detection $\langle\mathcal{B}\rangle < 0$ given the irreps (p, q) that display nonlocality detection with $n = 25$ qutrits.

Irrep	RCS λ	NNSD (Brody) ω	$\langle\mathcal{B}\rangle$
(8, 6)	4.863×10^{-7}	4.110×10^{-11}	−0.146
(12, 1)	7.643×10^{-8}	9.659×10^{-11}	7.548×10^{-10}
(11, 3)	5.245×10^{-7}	1.242×10^{-09}	−0.146
(10, 5)	2.383×10^{-12}	9.416×10^{-11}	−0.339
(14, 0)	7.440×10^{-8}	1.116×10^{-10}	−0.297
(13, 2)	5.358×10^{-2}	2.326×10^{-01}	−0.405
(12, 4)	1.300×10^{-9}	4.165×10^{-11}	−0.767
(15, 1)	1.893×10^{-6}	3.116×10^{-06}	−0.889
(14, 3)	5.978×10^{-8}	2.830×10^{-09}	−1.178
(17, 0)	4.521×10^{-7}	5.325×10^{-11}	−1.372
(16, 2)	1.435×10^{-12}	5.323×10^{-11}	−1.726
(18, 1)	1.667×10^{-9}	3.907×10^{-08}	−2.319
(20, 0)	7.494×10^{-8}	3.916×10^{-11}	−3.013

Supplementary Table III. Same as Supplementary Table II but for $n = 20$ qutrits.

value $\langle\mathcal{B}\rangle$, irreps that exhibit nonlocality (*i.e.,* $\langle\mathcal{B}\rangle < 0$) generically display a Poisson RCS/NNSD. Conversely, irreps where no detection of nonlocality is observed generically display a Wigner-Dyson RCS/NNSD. We observe this behavior for different number of parties n, starting from $n = 8$ qutrits (when the PIBI we use starts detecting nonlocality) up to $n = 32$ (beyond which a numerical analysis becomes computationally expensive). For example, in Supplementary Figure 2 and Supplementary Table III we also present the $n = 20$ qutrits case for comparison. This observation leads us to believe that for the Bell operator of the PIBI (1) with measurements yielding maximal nonlocality detectability, irreps (p, q) have an RCS fitted with $\lambda = 0$ as n goes to infinity, signalling integrability. As we have seen in the main text, this integrability is likely explained by the additional parity symmetry that occurs around such optimal measurements. Note that in both cases there are some irreps apparently contradicting this conjecture (see the orange points in Supplementary Figures 1 and 2). However, we attribute such occurrences to finite size effects, since for $n = 25$ or $n = 20$ the scarce number of energy levels (after removing redundancies due to the permutation invariance) results in coarse-grained RCS/NNSDs.

Unfolding the energy spectrum. – To compare the nearest-neighbor energy level-space distribution of different operators, it is desirable to normalize it appropriately. The procedure we follow takes the name of spectrum unfolding [1] and it consists of the following steps.

First, the energy spectrum is sorted so that the energy levels $\{x_i\}_i$ are in ascending order $\{x_1 \leq x_2 \leq \ldots \leq x_k\}$. Then, we compute the cumulative distribution function $I_x(E)$ counting the number of energy levels up to energy E. This is a discrete function, which it is convenient to interpolate with a continuous polynomial function $\tilde{I}_x(E)$. The goal is now to rescale the sequence $\{x_i\}_i$ into a sequence $\{y_i\}_i$, such that its cumulative function $\tilde{I}_y(E)$ is a straight line. This is achieved by inverting the function $\tilde{I}_x(E)$ and using it to rescale the sequence $\{x_i\}_i$. This spectrum unfolding ensures that the local density of states of the renormalized levels $\{y_i\}_i$ is unity. From the latter sequence we compute the nearest-neighbour energy level spacing as $s_i = y_i - y_{i-1}$, which are used to obtain the NNSD.

SUPPLEMENTARY NOTE 3: LOOKING CLOSER TO THE ANOMALOUS IRREPS

In Supplementary Figure 4 we show the RCS histograms for the anomalous irreducible representations that deviate from the otherwise observed Poissonian spacing distribution observed at optimal measurements in the remaining irreps detecting nonlocality. Namely, the cases $(p, q) = (9, 8)$ and $(15, 2)$ for $n = 25$, and $(13, 2)$ for $n = 20$. For each irrep, we present the RCS distribution obtained both from the measurement settings yielding maximal quantum violation and from randomly generated measurement settings. For the random measurements, we have selected instances whose RCS distributions are very closely fitted by $\lambda \simeq 1$, corresponding to clear Wigner-Dyson (GOE-like) behavior. We emphasize, however, that random measurements do not always yield $\lambda \approx 1$. Rather, they produce a distribution of fitted λ values, with $\lambda \simeq 1$ being the dominant case, as shown in Supplementary Figure 3.

Looking at the optimal measurement settings histograms, note that although the fitted RCS parameter is non-zero ($\lambda > 0$), the corresponding histograms do not exhibit a pronounced suppression of small spacings. In particular, the first histogram bin does not clearly display level repulsion, in contrast to the random-measurement cases, where level repulsion is clearly visible already at small spacings. This qualitative difference suggests that these spectra lie in a crossover regime between Poisson and Wigner-Dyson statistics, rather than exhibiting fully developed chaotic behavior. This strengthens our interpretation of the residual non-zero λ for the optimal measurements as a finite-size effect that might vanish as the irrep dimensions increase towards the asymptotic limit. On the other hand, random measurements generically yield Wigner-Dyson statistics. Hence our conjecture that, in the asymptotic limit, the Bell operators corresponding to measurement settings that maximally violate the Bell inequality are universally integrable within this class of Bell operators.

[1] F. Haake, S. Gnutzmann, and M. Kuś, *Quantum Signatures of Chaos* (Springer International Publishing, 2018).
[2] T. A. Brody, Lettere al Nuovo Cimento (1971-1985) **7**, 482 (1973).
[3] E. Bittner, H. Markum, and R. Pullirsch, arXiv:hep-lat/0110222 [hep-lat] (2001).

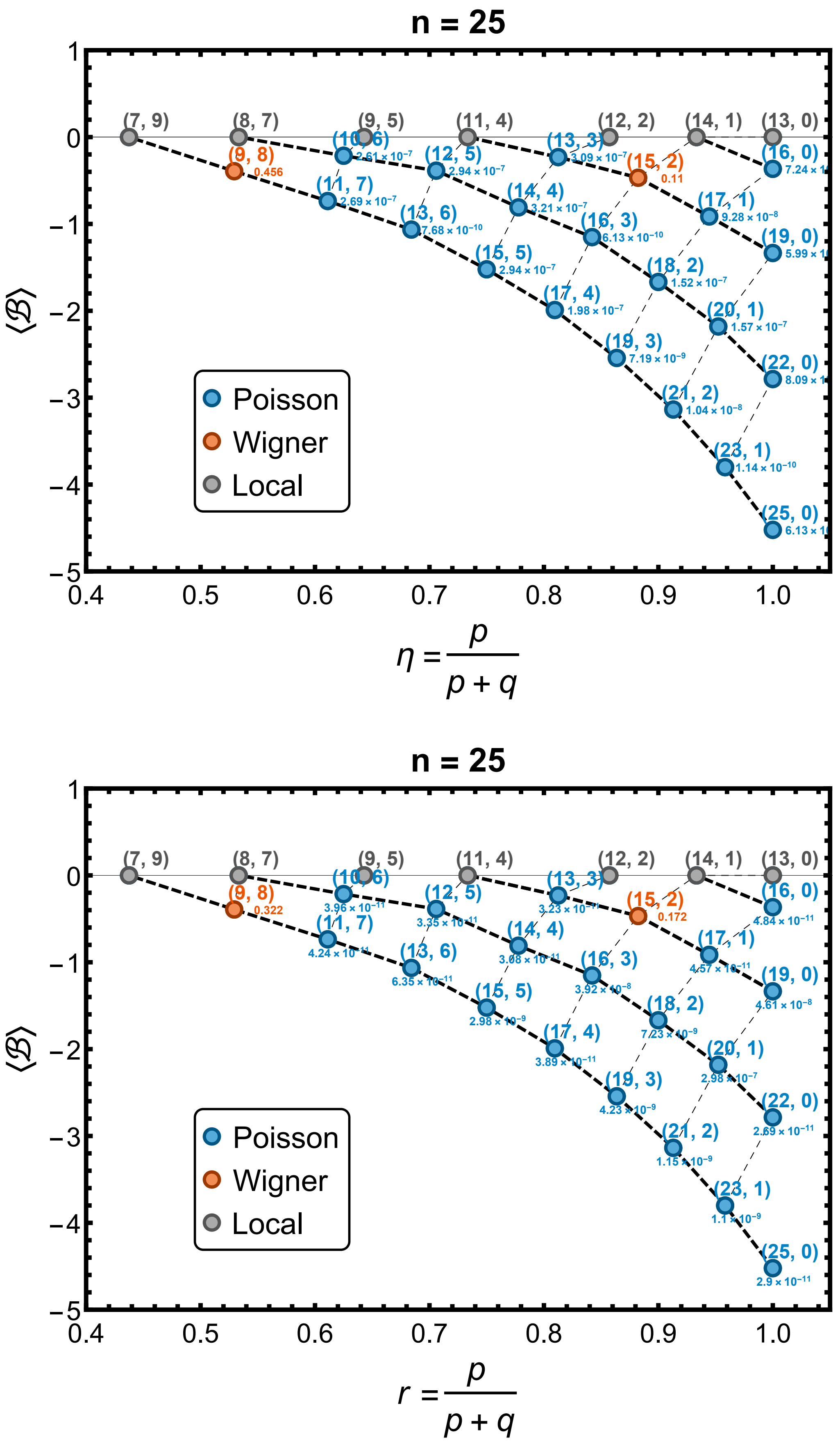

Supplementary Figure 1. (a) Same as the main text Fig. 2, now including the interpolating parameter λ fitted to the RCS histograms. (b) Nearest-neighbor level spacing (NNSD) analog of (a), showing the Brody interpolation parameter ω as small labels. All numerical values are listed in Supplementary Table II.

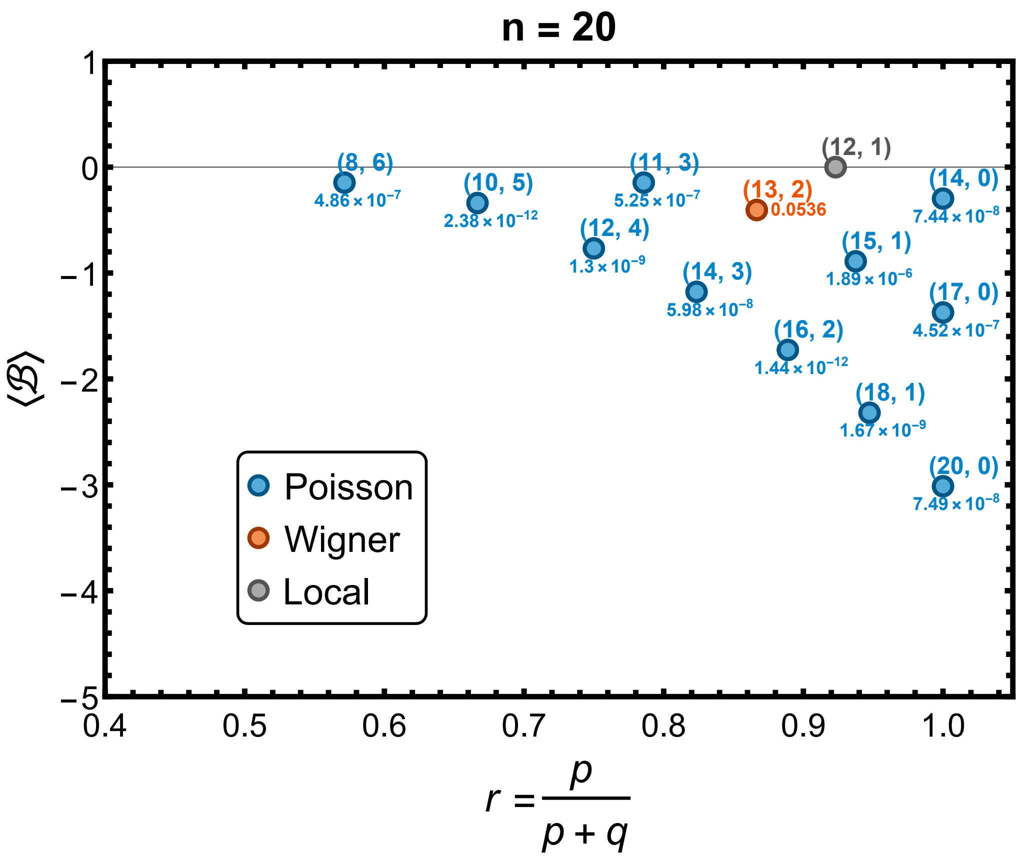

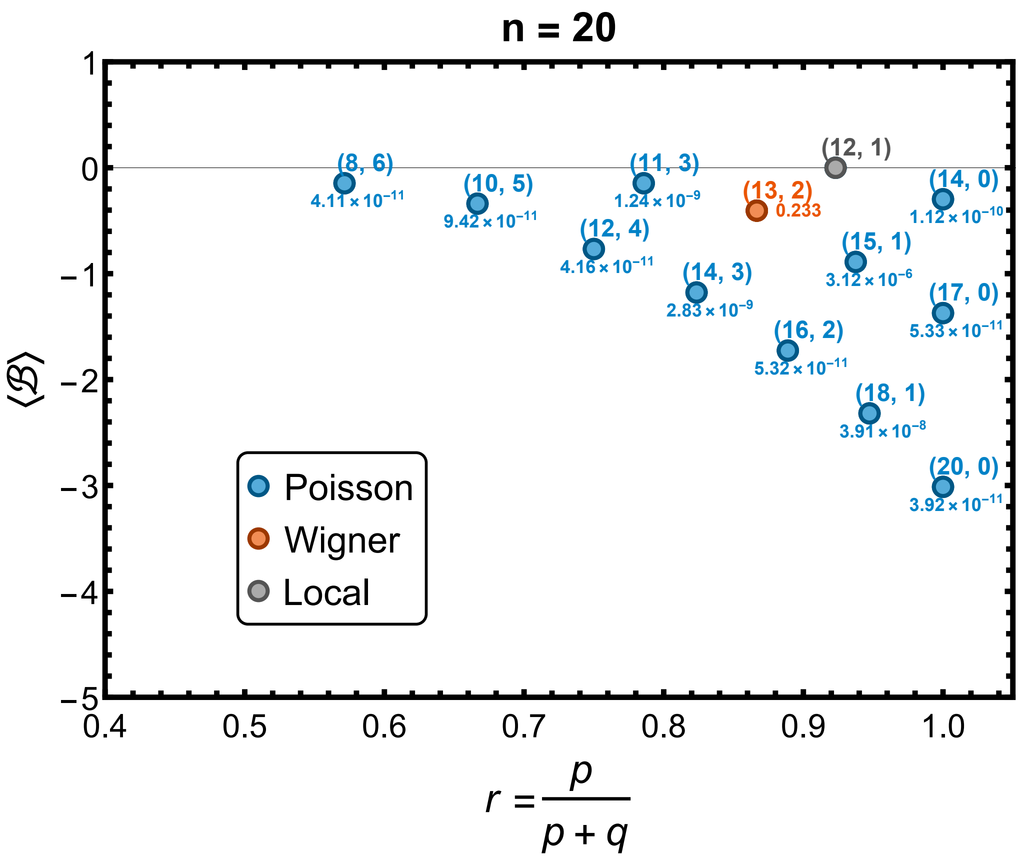

Supplementary Figure 2. (a) Same as Supplementary Figure 1 but for $n = 20$ qutrits. (b) Nearest-neighbor level spacing (NNSD) analog of (a), showing the Brody interpolation parameter ω. The data shown numerically in both plots is summarized in Supplementary Table III.

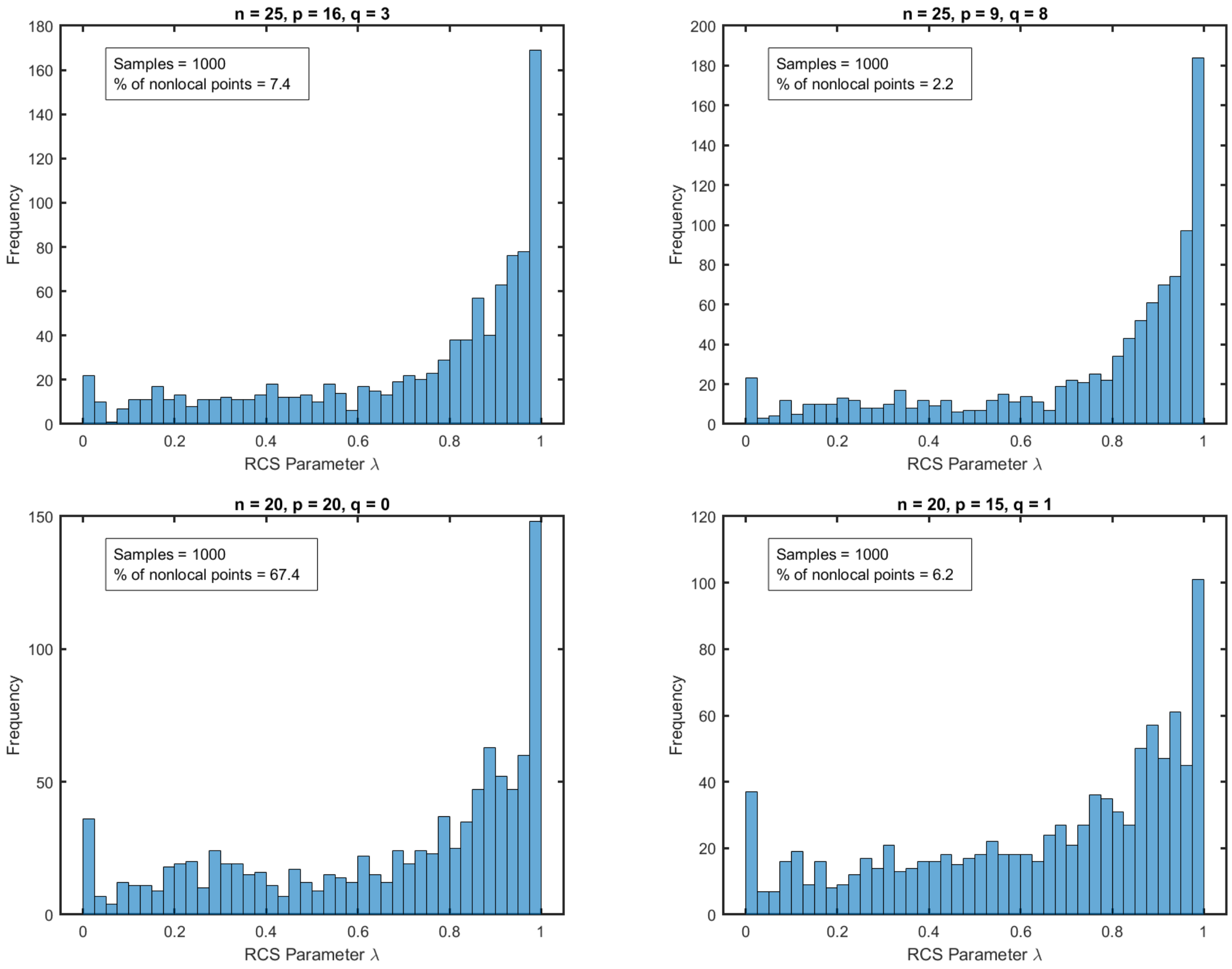

Supplementary Figure 3. More histograms for different irreps with $n = 25$ (top) and $n = 20$ (bottom), using 10^3 random projector samples per case, to reinforce that the trend observed in Fig. 3 of the main text is generic across various irreps. That is, all cases show a clear departure from the Poisson distribution, consistent with chaotic behavior in the Bell operator induced by random measurements.

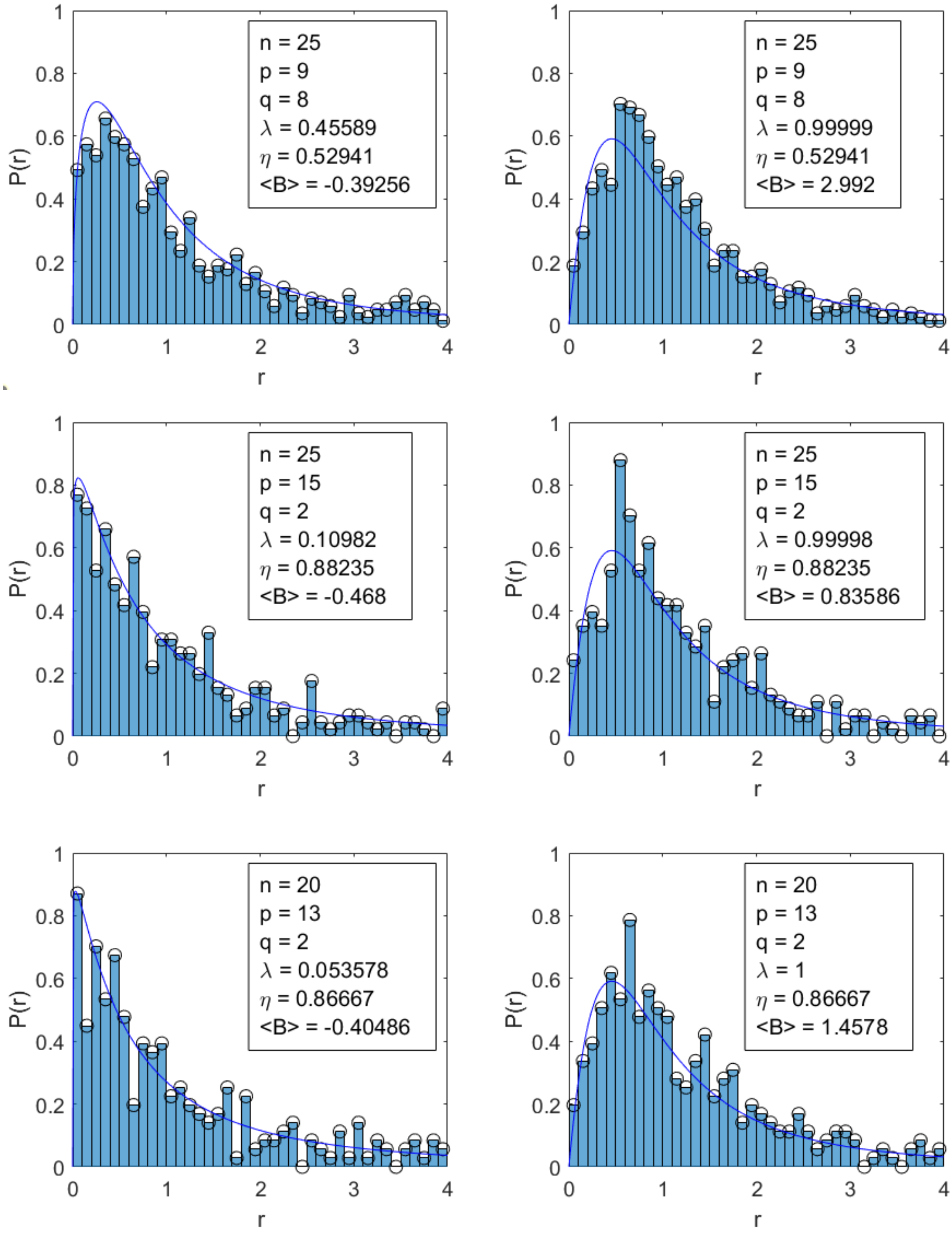

Supplementary Figure 4. RCS histograms for the anomalous irreducible representations $(p, q) = (9, 8)$ and $(15, 2)$ for $n = 25$, and $(13, 2)$ for $n = 20$. For each irrep, the left panel shows the spectrum obtained using the measurement settings yielding maximal quantum violation, while the right panel shows a representative case obtained from randomly generated measurement settings with an RCS fit $\lambda \simeq 1$. Although the optimal-measurement spectra yield a non-zero fitted parameter $\lambda > 0$, they do not exhibit a clear suppression of small spacings (noticeable in the first few bins) and therefore fall into a crossover regime between Poisson and Wigner-Dyson statistics. In contrast, the random-measurement spectra display clear level repulsion at small spacings, characteristic of Wigner-Dyson (GOE-like) behaviour. This observation supports our conjecture that, in the asymptotic limit, the Bell operators corresponding to measurement settings that maximally violate the Bell inequality are integrable within this class of Bell operators.
